# Mental health promotion in municipal settings – promoters and inhibitors in the implementation of ABC for good mental health in Norway

**DOI:** 10.1186/s12889-026-27493-z

**Published:** 2026-04-30

**Authors:** Tanja Plasil, Gudveig Gjøsund, Martin Inge Standal, Julie Marit Granlund

**Affiliations:** 1 Studio Apertura, NTNU Samfunnsforskning AS (NTNU Social Research), Dragvoll Allé 38 B, Trondheim, 7049 Norway; 2 Nasjonalt Kompetansesenter for Psykisk Helsearbeid (NAPHA), Trondheim, Norway

**Keywords:** Mental health, Public health, Settings, Health campaign, Organisational framework, Municipalities

## Abstract

**Background:**

Mental health promotion has become an increasing priority in Norwegian public health work, particularly in response to rising mental health challenges. The Act-Belong-Commit (ABC) framework was piloted in three municipalities in Trøndelag county, Central Norway, before being adopted as a national campaign in 2025. Despite its rapid rollout, little is known about how such public mental health campaigns can be implemented and their potential for creating health-promoting settings. In this article we therefore identify the inhibiting and promoting factors in the implementation of ABC and ask if ABC can create more mental health promoting settings.

**Method:**

This qualitative implementation study draws on semi-structured interviews conducted between May and October 2025 with 16 informants across four municipalities (two pilot, two non-pilot) in Trøndelag county, Norway. Data were analysed using thematic analysis, identifying five themes: background and timing, organisational structure, actions and activities, communication and collaboration, and material and evaluation.

**Results:**

Promoting factors included timely uptake, leadership support, cross-sectoral public health teams, and integration into existing strategies and work processes. Inhibiting factors included organisational instability, overreliance on event-based activities, insufficient collaboration with volunteer organisations, and a consistent absence of systematic evaluation across all municipalities.

**Conclusions:**

ABC, when implemented successfully, offers a flexible, recognisable, and accessible framework that can contribute to healthier settings for both employees and citizens. ABC is most successful when integrated into already existing systematic public health strategies. Successful implementation requires structural anchoring, long-term commitment, and robust evaluation mechanisms to ensure sustainable impact.

**Supplementary Information:**

The online version contains supplementary material available at 10.1186/s12889-026-27493-z.

## Introduction

Mental health promotion seeks to enhance the mental health of the population in general or of specific groups which might be at greater risk of mental illness [1–3]. Mental health promotion can have long-lasting positive effects if grounded in multi-component, multi-setting, participatory programmes rather than single-issue or clinically focused approaches [4]. According to the World Health Organization Ottawa Charter [5] health promotion should build on five key strategies, namely healthy public policy, building supportive environments, strengthening community action, developing skills and reorienting public health services. Even though the Ottawa Charter does not prescribe "public health campaigns" specifically, the Charter's emphasis on developing personal skills, empowerment, and access to information has long been used to justify and guide campaign-based health promotion.

However, implementation of public health campaigns can be difficult, even when the campaigns themselves are proven to be effective [6]. Such difficulties are commonly due to poor coordination, limited resources and funding, lack of stakeholder input, and a lack of focus on evaluating the implementation. Conversely, political support, long-term and strategic thinking, sufficient resources, community involvement, and iterative learning processes are facilitators for success [6–9]. It is therefore important to gain insights into how mental health promotion campaigns are planned and executed in order to gain knowledge for future implementation processes [10]. In this article we will present implementation experiences from a particular mental health promotion campaign, ABC for Good Mental Health, in central Norway, to identify promoters and inhibitors for successful implementation processes.

In Norway, municipalities are required by law to have a long-term and systematic approach to health promotion that involves cross-sector collaboration to prevent illness and disease and to reduce social inequalities in health (Lov om folkehelsearbeid 2011/2012 – Public Health Act). Public health efforts have traditionally focused on the physical health of the population, but due to rising mental health issues, especially among young people [11, 12], maintaining good mental health among the population has become a focus in the systematic public health work in Norway (e.g. [13, 14]). In a report on mental health in public health work, the Norwegian Directorate of Health stated that "both mental disorders and mental health are important; psychosocial perspectives are central when we work with causes and outcomes in public health work" [15].

One strategy for mental health promotion that has been used in Norwegian municipalities is the Act-Belong-Commit (ABC) framework [16]. The framework shifts the focus away from mental illness towards mental health and wellbeing, takes it out of the healthcare sector and makes it "everybody's business" [17, 18]. ABC encourages the population to actively engage in improving their own mental health by doing something active and meaningful together with others, as research shows that increased activity, degree of belonging, and extent of engagement were associated with the absence of anxiety and depression symptoms [19, 20]. ABC has a dual-aspect approach, meaning that it is both a public health campaign and an organisational framework that can be used in different settings such as voluntary organisations, municipalities, businesses, hospitals/clinics, and schools, and among different populations such as immigrants, children, or the elderly [16]. It furthermore offers a toolbox of practical instruments and resources for improving mental health, including cards, bingo, posters, guidelines for performance appraisals, and videos. ABC was first implemented in six pilot municipalities in Western Australia in 2006 [16] and then spread to Denmark [21, 22] Finland [23], and Norway [20, 24].

From an organisational point of view, the setting where public health promotion is advocated and implemented plays an important role. According to the WHO, a setting is a "place or social context in which people engage in daily activities in which environmental, organizational and personal factors interact to affect health and wellbeing" ([25]: 30). Public health campaigns can be implemented in different settings and among different populations (defined by, for example, age, gender, health status, location, socio-economic or cultural criteria). Poland et al. [26] argue that settings are more than simply locations, but that they are both the medium and product of human social interaction. Mental health promotion aims to make these settings healthier for those interacting within them [27–29]; Dooris [30] argues that a healthy settings approach helps to shift the focus from "the deficit model of disease to health potentials inherent in the social and institutional settings of everyday life" (p. 29). Furthermore, it is important to have a "whole systems approach" whereby health "becomes an integrative goal of the organisation regardless of whether the organisation is a workplace, a school or a business" [30]: 30,based on [31]) or, as in this research, a municipality. This whole systems approach is echoed in the supersetting approach, which advocates for a broad, holistic, and coordinated approach when implementing public health campaigns and initiatives [32]. "The supersetting approach is an intervention strategy whereby coordinated activities targeting a common overall goal such as improved health in a population group are carried out in a variety of different settings and involving a variety of different stakeholders within a local community" [32]: 3). Supersettings aim to achieve synergistic effects by carrying out activities for different populations in a coordinated manner within diverse settings. They are the most complex strategies for public health promotion.

Even if this article is an implementation study, we will not only investigate the promoters and inhibitors for implementing a public mental health campaign such as ABC but also ask ourselves whether ABC can promote healthier settings for mental wellbeing and whether participating municipalities could potentially become a supersetting. In Norway, the central region of Trøndelag county took the lead in implementing the ABC campaign and framework through a pilot project in 2024, in which three municipalities received extra funding to roll out ABC broadly (Trøndelag [33]). Soon after, it was decided by the Ministry of Health that ABC should become a nationwide campaign. However, little is known about how ABC should be implemented and what impacts successful implementation in different settings. It is therefore particularly interesting to examine the implementation process in the pilot municipalities, not only from a Norwegian point of view but also more generally, as research on ABC tends to focus on the effects [34, 35] of the campaign rather than the process of implementation itself.

In this implementation study we therefore aim to answer the following question: *What are the promoting or inhibiting elements in the implementation of ABC for mental health, and what is ABC's potential for creating more health-promoting settings?*

In this article we will thus not only investigate the promoters and inhibitors for implementing a public mental health campaign such as ABC but also ask ourselves whether ABC can promote healthier settings for mental wellbeing.

## Method

### Study background

Following the Public Health Act of 2012, Trøndelag county in central Norway implemented two public health measures: a model to work systematically with public health to develop, implement and evaluate effective health-promoting measures (Trøndelagsmodellen for folkhelsearbeid, [36]) and Folkehelsealliansen i Trøndelag (FiT), a network of non-commercial actors collaborating for the improvement of public health in the county, which soon expanded its focus from physical to mental health [37]. Soon after its establishment, FiT started focusing on ABC as a health promotion campaign.

Thus, Trøndelag county started actively working with ABC in 2019. In 2024, three pilot municipalities received funding and additional support to implement ABC. In 2025, the Directorate of Health launched a national campaign, and eleven county councils received grants to implement ABC in their municipalities. Implementing such campaigns can be challenging for municipalities, and this article is the result of a research project aimed at gathering practical and applicable information on the implementation of public health campaigns, using the ABC for mental health campaign as a case study, and how such campaigns can be used in municipalities.

### Data collection and participants

The data presented in this article are based on an interview study conducted between May and October 2025. As this was exploratory research, we conducted semi-structured interviews, which offer the possibility to adapt the interview during the process, thereby generating richer data material [38, 39]. We included two1 of the three pilot municipalities. During our first interviews it became clear that other, non-pilot, municipalities had also begun to implement ABC either within their own organisation or outwards towards the public. We therefore decided to include two non-pilot municipalities in the sample to examine whether the additional funding and support provided to the pilot municipalities influenced the implementation processes.

In total we interviewed 25 people (in four interviews we had two interviewees; the rest were individual interviews) in the project. In this article we only include data from our interviews with eleven municipal employees and five county employees, as interviews conducted with private businesses or other non-municipal workplaces were not relevant for this article. Within the municipalities we interviewed public health coordinators (3), leaders in (mental) healthcare services (3), HR personnel (1), psychologists (2), and staff at the mental healthcare services (2). Within the county we interviewed advisors (3), a project leader (1), and a researcher (1).

All informants received written information prior to the interview and were asked for their consent at the beginning of the conversation. Interviews lasted for one hour, were recorded, and transcribed verbatim. All interviews were conducted in Norwegian; citations used in the article are translated from Norwegian into English by the authors.

### Data analysis

This study had an inductive approach, using data as the starting point to develop different themes and topics. We used thematic analysis to analyse the data material from the interviews [40–42]. Thematic analysis as a method was considered appropriate for this study as it is "a method for identifying, analysing and reporting patterns (themes) within data" [40]: 79). The transcribed interviews were repeatedly read, emerging thoughts and themes were noted and discussed in the research group before coding the results into the different themes presented in the results section. The five themes within the implementation process of ABC in the municipalities are: 1) implementation background: approach, timing and societal challenges, 2) overall and specific organisational structure, 3) areas of implementation: actions and activities, 4) communication and collaboration, and 5) material and evaluation. In Table 1 we give an overview of some of the relevant findings categorised into the five themes and four participating municipalities.

**Table 1:** Overview of ABC campaigns

	**Municipality 1**	**Municipality 2**	**Municipality 3**	**Municipality 4**
Backgound				
Pilot	Yes	Yes	No	No
Why?	Mental health of children/adolescents; loneliness; elderly people	Improvement of mental health and quality of life; refugees (to a lesser extent)	Municipal restructuring following some dramatic events	High sick-leave rate in municipality (especially health-care services)
Organisation				
Direction	Top down	Top down	Top down	Top down
Responsible	Public health coordinator	Public health coordinator, also working as communication officer in town hall	Public health coordinator (working in a 20% job)	HR coordinator
Supporting team	Cross-sectoral public health group (existing)	Cross-sectoral public health group (existing) and a new ABC-group	Tries to build a public health group	Existing HR group (now ABC group)
Actions and activities				
Kick-off event	No not one event	Large event for municipal staff and population at the same time	One with volunteer sector, two with private business sector	During a unit leader meeting
Focus on whole population	Yes	Yes, in phase 1, not anymore	Yes, that is the plan	No
Focus on municipality’s work environment	Yes, but depends on unit (yes for mental health unit)	Yes, in phase 2 (focus on town hall)	Yes, a little bit	Yes, main focus
Implemented in mental health services	Yes	To a certain extend but not explicitly	No (due to situation)	Not specifically
Communication and collaboration				
Volunteer sector involved	Yes	Not enough	Yes, a little	No
Private business sector involved	No	Yes, a little	Yes, a little	No
Communication channels	Public events, radio, information screens, municipal webpage, social media	Public events, municipal webpage, social media	Three events, internal communication (emails)	Staff meetings and internal communication (email, intranet)
Material and knowledge				
Training	No (got help from Red Cross)	No (got help from county)	No (got help from Nav)	No (got help from Nav)
Use information from	County website and ABC network	County website and ABC network	County website and Nav	County website and Nav
Evaluation done	No	No	No	No

## Results

### Hearing and doing – implementing ABC in four Norwegian municipalities

As the table above shows, the four participating municipalities each had a different approach in the implementation process depending on their specific needs and local circumstances. Here we present our findings grouped under the five themes that emerged from the data.

### Implementation background: approach, timing and societal challenges

Each of the four municipalities participating in the research had its own reasons for implementing ABC. Both pilot municipalities cited growing mental health issues and loneliness among both young people and the elderly, and a need to address mental health problems elsewhere than solely through public health services. The two non-pilot municipalities had more particular reasons: municipal restructuring following dramatic events in one, and high levels of sick leave in the other. Even though each municipality had a different underlying challenge, all pointed out that ABC was seen as the most appealing solution to their problems. One informant from a pilot municipality described the process as follows:

ABC is so easy and logical, […] wrapped very nicely in that simple framework. It was presented in such a nice and colourful way that it was impossible to dislike it. After having heard a presentation about ABC, the municipal director decided on the way home that the municipality should be part of the pilot.

It was those municipalities that were most interested, or *pushy* as one county informant called it, that received funding for establishing a pilot. Afterwards, the Norwegian Ministry of Health made ABC a national campaign, even though, as our informants stated, ABC was not the only campaign or framework for improving mental health that the municipalities were encouraged to implement. However, according to one project leader and the advisors from the county: “*ABC is easier to remember than other public health campaigns as it consists of three simple steps symbolised by three letters*.” Furthermore, as was pointed out by the county project leader, ABC, unlike other campaigns, has "both a hearing and doing approach". The hearing aspect is pointing to the campaign dimension of ABC, whilst the doing aspect meant implementing ABC as a framework within the municipality.

ABC's national spread also depended on the right timing, as one informant from the county put it: "*I am sure there are many campaigns and tools for improving public health in many drawers – it just needs the right time and interest to get them out*." Hence, the ABC campaign, which FiT had begun to roll out as early as 2019, finally gained momentum and was adopted nationally because its simple message and dual approach seemed a timely match for diverse mental health challenges Norwegian municipalities are facing today.

### Overall and specific organisational structure

In all four municipalities, the decision to implement was top-down, but only one pilot municipality reported that this led to some resistance among unit leaders, without elaborating on the specifics. In general, according to our informants, broad acceptance was achieved by the initial enthusiasm of the municipal senior leadership, which spread down to the unit managers who could then convey the message to their employees. In all municipalities, the initial top-down approach (often driven by municipal managers) was filtered through either the public health coordinator or HR services to the general population and/or municipal units. All informants stressed the importance of leadership support for a public health campaign such as ABC to succeed. However, continued support is not always guaranteed. In one of the pilot municipalities, "*the political agenda changed, extra resources stopped and top-level leadership changed course*," one of the informants told us. Here, despite the initial success of a broad campaign, the use of ABC was moved from being a citizen-oriented public health campaign to a framework used within the municipal workplace only. This is not only an example of how leadership might change course, but also a reminder, as all informants told us, that public health campaigns should not be thought of as a project, but must be embedded in existing public health work and connected to other plans and strategies, such as public health, volunteer or social strategies, and HR plans etc.).

Within the two pilot municipalities, ABC was grounded in well-established, cross-sectoral public health teams which included members from different municipal units such as healthcare or culture, and within one of the non-pilot municipalities, ABC was anchored within the municipal HR team. According to both pilot and non-pilot municipalities having an existing group made it easier for the coordinator to find partners for implementing the ABC framework rather than working alone. This was vital for success: "*without an already existing group our public health coordinator would have sat rather sad and alone*," one manager told us. Furthermore, according to this informant, it was important that both the coordinator and the group were "*situated in a central administrative unit, not out in the practical services*," and that they carried some weight in the municipal hierarchy. According to one public health coordinator, it was important to understand ABC as a lasting change in approach towards mental healthcare and a mode of working rather than a short-term project, and that rather than being something additional, the ABC framework and message had to be embedded in everyday work processes, work culture, and annual plans.

The two non-pilot municipalities also stressed the importance of incorporating ABC into existing work plans; however, this applied on a different level. In one non-pilot municipality, ABC was implemented exclusively within the municipality as a workplace and not broadly towards citizens. According to our informants from HR, ABC made it easier to talk about mental health in the work environment, and even though the concept sometimes merely resembled "*common courtesy and good manners*," using the ABC brand placed actions within a recognisable framework. Therefore, as our informants from HR pointed out, it was important to systematise the use of ABC beyond singular team-building events by incorporating it into the yearly planning, so that the message would be repeated during meetings and conversations.

The other non-pilot municipality lacked an existing public health team or strategy. This made it difficult to implement ABC from the outset. Further difficulties arose due to the restructuring of the municipal units and a renovation of the municipal buildings, which meant that many employees were located in temporary offices. Still, the municipality decided to implement ABC not only as a public health campaign, but also as a tool in the mental health services and as a framework for improving the working environment within the municipality itself. However, without a functional support group or sufficient resources, and facing an ongoing difficult situation, it was hard for the public health coordinator to deliver: "*there might not be as many in the ABC group as I would have liked, but those who are here are very engaged. […] We start with what we have*." Therefore, in this municipality, the coordinator chose to build step by step on the available human and financial resources rather than going all out as the pilot municipalities had the means to do.

All examples show that for a successful implementation, initial enthusiasm and a convincing message are not enough. For ABC to become a success, either as a public health campaign or as a health-promotion framework within the municipal workplace, continuous work and leadership support are essential, as is having a dedicated team and a systematic approach towards mental health within the municipality.

### Areas of implementation: actions and activities

When discussing the implementation process of ABC, informants very often described activities that had been organised. Depending on the reasons, available resources, and focus of the ABC implementation, each municipality developed and executed different actions and activities. Municipality 1 (pilot municipality) used ABC in three ways. Firstly, ABC was used in a public health campaign aimed at the general population through large events such as a "Join-In-Day" at the municipal cultural centre, where different cultural and voluntary activities were presented. Furthermore, the ABC message was spread through publications on the radio, information screens, and the municipal (social) media, and through more regular, already existing events aimed at specific groups like for example, making ABC part of the mandatory introduction programme for refugees, presenting it during elderly cafés, or using ABC to work with the classroom environment at the local school. According to the public health coordinator, it was important that the activities were connected to already existing municipal events and that the message would be included in all municipal events to guarantee branding and recognition. Secondly, ABC was implemented to improve the working environment in some of the municipal units, for example in mental healthcare. ABC tools were used in performance reviews and during meetings to encourage discussion about staff mental health and work culture. Thirdly, ABC was used as a tool in the mental health services. According to our informants from the mental health services, ABC's focus on doing something active with others was not a new concept; however, placing these recommendations under one common denominator provided a useful, easily communicable, and recognisable framework. ABC as a framework and activating message was used as a supplement to other treatments and therapies.

In the other pilot municipality, ABC had not been implemented in the mental healthcare services. According to one informant working in the services, this was because ABC's message was "*more beneficial for those who do not work with mental health professionally to use the ABC message, as it is easy to sell to the public to help them reduce mental health problems*." Therefore, this municipality chose to focus more on ABC as a positive campaign rather than as a tool in mental healthcare, making ABC a well-known brand by "*including ABC in most of the municipal events*", as the public health coordinator told us. The pilot was launched with a large kick-off event for both municipal (mental) healthcare employees and the general population. Other promotional activities took place during a comedy festival, the municipal information week, a job fair, a large sports event, and the World Mental Health Day event. ABC was furthermore promoted through the municipal website and social media.

Both pilot municipalities had the ambition, political will, and resources to roll out ABC broadly. The two non-pilot municipalities took a different approach. Due to the circumstances, lack of resources, and restructuring, one of the non-pilot municipalities could only take small steps at a time, trying to use ABC as the common thread to establish more systematic public health work and to establish a public health group. Therefore, one ABC event with the voluntary sector and two with private businesses were organised. Furthermore, according to the public health coordinator ABC was seen as a useful, non-threatening tool to "*make for a better work environment in challenging times*." However, due to limited resources and the challenging reorganisation, ABC had not yet been implemented in the mental health services.

The other non-pilot municipality had opted to use ABC solely as a framework for a healthy work environment within the municipality itself. After a kick-off meeting with all unit and senior leaders present, an ABC group was established, led by HR services. Actions taken were mainly social activities such as a bowling evening, a best-ABC-action competition, and regular Friday coffee meetings. These may seem like small things, but in an environment where people work shifts, such actions could make a difference, according to our informants. Again, it was stressed that ABC should not create additional work but rather give employers and employees some tools and a framework to address the topic of mental health at work and to work collaboratively on improving the work environment. HR also pointed out that "*it is important to try and feel ABC first yourself before you go out and try to convince others*," thereby recommending trying ABC internally before attempting to reach the wider public.

Across the four municipalities, ABC was implemented through a wide range of actions, from large campaigns to workplace initiatives to small steps. This shows that the framework has the ability to be adapted to context, resources, and priorities.

### Building on existing communication and collaboration

All municipalities described ABC as "*an unproblematic and non-threatening approach towards mental health work,*" as this informant from a non-pilot municipality called it. Most informants agreed that ABC's central message of doing something meaningful together with others, and its concept of making mental health everybody's business, was meaningful and easy to convey. According to one informant from the mental health services, ABC made people aware that "*we all have a mental health, and, like our physical health, it changes through life; it can be better or worse*." However, one informant from the healthcare services was more sceptical about the "easiness" of the ABC message, stating that "*you do not decide yourself how your mental health will be. You can have bad experiences; you grow up a certain way,*" and that ABC could lead people "to feel that this is yet another thing they failed to do compared to others."

This scepticism represented the minority opinion in our interviews. The majority agreed that, on the one hand, the positive and concrete message of ABC made it easy to communicate beyond the mental healthcare services to the whole population; on the other hand, informants pointed out that the message could be seen as too simple, almost banal, as something that is neither new nor revolutionary. One leader in the mental health services put it thus: "*ABC is not a Hallelujah, it does not save the world […] but it is a good starting point*." According to all informants, ABC should be a supplement to other measures used in systematic public health work to enhance mental health among the public, in the workplace, or in the treatment of patients and not a single solution. However, all acknowledged that ABC indeed offered a coherent framework that is useful for enhancing mental wellbeing among both municipal staff and the wider population.

All municipalities had to acknowledge that finding and engaging those who were lonely or already had mental health issues was difficult through the ABC framework, and they had not yet found strategies to do so, as one leader in the mental health services explained: "*I do not know where they live. I do not know how to reach them*." Here, many informants hoped that voluntary organisations could play an important role. In the first phase of the ABC campaign in Trøndelag, volunteering played a central role. Our informants from the county told us that the Red Cross developed large parts of the ABC information and implementation material used by the county and the participating municipalities and organisations today. However, all public health coordinators we interviewed had to acknowledge that, even though they tried to include more voluntary organisations in the campaign, the collaboration with them could have been better. These organisations were seen as crucial for the continuation of ABC, both for spreading the message and for providing activities once additional funding and municipal resources came to an end. According to several public health and HR personnel, maintaining ABC as a framework, both towards the public and in the workplace, required the message to be repeated continuously, or as one put it, "*you have to come out with information in small doses all the time*." The question is whether these small doses will continue once the funding has stopped.

While most informants viewed ABC's simple, positive message as a useful and accessible framework for promoting mental wellbeing, many also noted its limitations, warning that it can feel overly simplistic, risk burdening individuals, struggle to reach those most in need, and ultimately requires ongoing, collaborative effort (including voluntary organisations) to remain meaningful and effective.

### Material and evaluation

It is noteworthy that, according to our informants, none of the municipal staff received any formal ABC implementation training. Most initially heard about the framework through a presentation and then used county advisors and other ABC promoters for an initial information meeting, workshop, or kick-off event within their own municipality. Afterwards, material from the county's ABC website was used to learn about and implement ABC within their own organisation making use of centrally provided campaign material, such as bingo cards, posters, or conversation guidelines. While this was not seen as problematic by either municipal or county informants, some HR employees and public health coordinators would have liked to see additional material in the future, as the supply on the website was limited.

However, the municipalities were most self-critical about the fact that no systematic evaluation of the implementation process had been conducted before ABC became a national campaign. All four municipalities acknowledged that they had not conducted a thorough evaluation process, and that this was a weakness both for the continuation of ABC within systematic public health work and for mental health promotion in the workplace.

Table 2 presents an overview of the promoters and inhibitors we identified before we go on to discuss our findings in the light of our research question: *what are the promoting or inhibiting elements in the implementation of ABC for mental health, and what is ABC's potential for creating more health-promoting settings?*

**Table 2:** Promotors and inhibitors for successful implementation of ABC

Element	Promotor	Inhibitor
Background	Right timing	Roll out before evaluation
	Hearing and doing approach	
	Take initiative to get funding	
Organisation	Initial and continuous leadership support	Resistance by unit management
	Cross-sectoral public health teams in central administrative locations	Reduction or shift in leadership support
	Previous systematic public health strategies	Restructuring and relocations
	Integrate in existing strategies, work processes and operational plans	Lack of systematic public health work and public health teams
		Dependence on individual commitment
		Treat it as a project
Actions and activities	Connect to existing activities and events	Need for additional resources to organize activities
	Multi-setting approach	Focus on fun events and activities rather than structural work
	Flexibility in the framework	
Communication and collaboration	Simple, positive, and easy to communicate messages	Being overly simplistic
	Collaborate with others	Expect and/or promise too much
	Collaborate across sectors and municipal units	No making enough use of other sectors and organisations
	Be aware of limitations of an approach	One size-fits all approach
Material and evaluation	Use previous and easily accessible material	Not enough educative or practical material
	Build up knowledge	Failure to evaluate the implementation process
	Evaluate before taking further steps	

## Discussion

### ABC does not save the world – but it can contribute to make it better

Based on the results, we first present promoting and inhibiting elements affecting this municipal health promotion campaign implementation before discussing the potential of ABC specifically for creating more health promoting settings.

### Promoting and inhibiting factors for the successful implementation of ABC

Barry [10] reminds us that it is important "to understand what actually happens during programme planning and delivery" [10]: 240), and many of the factors we see in the present study, such as coordination and organisation, management support, resources, collaboration, long-term thinking, and iterative learning processes through evaluation, have been identified in other recent studies. [6–9]. Even though the results describe a particular mental health campaign and framework, the findings can be relevant for the implementation processes of other campaigns as well. Within the five themes in this study, several promoting and inhibiting factors for the implementation of ABC can be identified. When looking at the background for implementation, timing was important both at the municipal and county level. When describing decision-making processes, it was pointed out that the moment to bring forward a mental health campaign was chosen due to rising mental health issues, organisational strain, or sick leave challenges. As shown above, timing is important when putting an idea, whether it has been sitting in a drawer or applied elsewhere, to work in solving the problems of the moment. It is also important that the chosen approach resonates well with senior leadership and other decision-makers, as success in health promotion work has been proven to depend on the attitudes and actions of municipal managers and senior officials [9, 43]. ABC's success over other mental health campaigns was due to its dual approach (campaign = hearing; framework = doing), which appealed to leadership as it combined messages with tools to address the challenges municipalities are facing. Another promoter for successful implementation is an assertive approach to securing funding, as it was noted that those who were "pushy" were rewarded.

Several inhibitors and promoters can be found in the overall and specific organisation of implementation processes. Clear leadership support is essential for both initial and continued success, as management enthusiasm lends legitimacy, opens up the use of resources, and can filter down to the units. However, even if initially successful, once leadership and political support diminish, implementation processes can come to a halt or be redirected, as happened in one of the pilot municipalities.

As found in similar studies (e.g. [7], organisational structures and embedding mental health-promoting projects into existing municipal structures is probably the most important factor for success. Municipalities that had previously worked systematically with public health promotion or health promotion in the workplace, and that already had well-established, cross-sectoral public health or HR teams, had structures in place to facilitate collaboration across sectors and thereby ease implementation. It was also important that these teams were located centrally within the administrative units, making them more accessible, and lending them legitimacy and the necessary influence. Where resources were limited, systematic public health work was a novelty, and no cross-sectoral team existed (as in one of the non-pilot municipalities) implementation was made more difficult. Municipal restructuring, temporary offices, and general instability further hindered the ability to implement ABC effectively. However, building on gradual implementation ("start with what we have") still allowed positive progress. This also demonstrates that it was important to integrate public health campaigns into already existing strategies, work processes, and operational plans, rather than implementing them as something "extra" or "on top of other things," which could potentially cause resistance. Furthermore, insufficient anchoring of new approaches within existing structures and processes could make continuation too heavily dependent on individual coordinators' commitment, leaving a campaign at a project level rather than as an integrated framework.

When it comes to areas of implementation through actions and activities, these were dependent on the initial approach of the implementation process, focusing either on the public or the workplace. In any case, it is beneficial to think strategically and to connect new campaigns to already existing activities and events, as this reduces the amount of additional work and embeds them in already existing structures [8]. As shown above, it was important for the ABC campaign to be visible at existing events directed towards public health, as this strengthens branding and recognition. Using a campaign across multiple settings or units further strengthens recognisability. Here it helped that ABC is quite flexible and could be used in many different contexts and through many different actions and activities. However, this flexibility could also be a weakness, as all municipalities focused heavily on organising "fun" activities (different events, activities, and actions were often emphasised in the interviews) rather than embedding the framework deeply into work processes and structures. As events and public activities are often dependent on additional financial and personnel resources, reliance on events poses a potential threat to continuity.

To ensure a more structural approach to implementing public health frameworks such as ABC, knowledge-based management and training of municipal personnel are important [7]. While none of the participating municipalities reported that managers or staff had followed a particular training programme (see Table 1), they had received support from the county and other organisations, and the county website offered many tools that could be used in practice.

When promoting mental health through (mass) media campaigns, concerted communication strategies and continuous commitment are essential [8]. ABC's simple, non-threatening, and positive approach made the core message easy to communicate to many groups and in many settings. However, there can be a downside when a message becomes too simplistic, banal, or commonplace and is perceived as something that has been done before, as this curbs engagement and enthusiasm for new frameworks. There is also a danger that a simple message might be understood as a quick fix or one-size-fits-all approach. The flexibility of ABC made it possible to use it in many settings and contexts, which works in favour of any campaign and framework; however, there is a danger that the simplicity of ABC becomes an approach that favours those who can actively participate in society, overlooking socio-economic differences and personal histories. This can, and did, lead to difficulties in reaching those who might need the campaign the most. To counter this shortcoming, it is necessary to repeat the message in different settings to widen the chance of reaching people, and to work together with other sectors and organisations, particularly voluntary organisations. According to Leinonen and Syväjärvi [9], health promotion strategy work requires collaborative methods. Collaboration with voluntary organisations in particular has previously shown strong potential for making ABC a lasting success [44]. However, all municipalities reported that they had not made sufficient use of this potential. Other campaigns should learn from this shortcoming and ensure that they invest enough in societal anchoring through collaboration.

Several publications (e.g. [6, 8]) identify monitoring and evaluation as important factors in processes of upscaling health-promoting projects. Moreover, several models for public health interventions point to the necessity of grounding implementation in evidence and evaluation processes (e.g. [36]) before upscaling. This step, pausing to assess and, if necessary, making adjustments, ensures that new public health strategies are on the right track before further effort is invested in them. This has not yet been done with the ABC campaign in the four municipalities, making the implementation vulnerable to misalignment with reality due to a lack of overview, critical reflection, and measurability.

In this section we have listed inhibitors and promoters based on the specific experiences of ABC in Trøndelag county; in the next section we discuss ABC's potential for building mental health-promoting settings.

### Potential of the ABC framework for creating mental health promoting settings

Barry et al. [2] emphasise that mental health promotion should take a whole systems approach, and this is what the pilot municipalities had in mind when implementing ABC both as a campaign and a framework, with the ambition to use it in multiple settings, among multiple populations, and through multiple channels and activities. Faced with mounting (mental) health problems within Norwegian municipalities, ABC, with its focus on improving mental health outside of the municipal healthcare services, seemed timely and opportune to several municipalities in central Norway. We now discuss, based on the experiences of our informants, whether ABC as a campaign and framework could potentially help create healthier settings for municipal workplaces and the general population.

Most of the informants experienced ABC as potentially contributing to the creation of healthier settings. When it comes to "shifting the focus from the deficit model of disease" [30]: 29), ABC, with its focus on prevention and potential rather than disease and deficits, contributed positively. According to the literature on ABC, combining the coherent, positive message (hearing) with concrete measures aimed at improving mental health (doing) across multiple settings, directed towards different populations simultaneously, can help achieve lasting behaviour changes among a municipality's inhabitants and employees [19], Haug et al. [20]). According to the informants in our study, the ABC framework helped to systematise public mental health work in several ways. As mentioned above, ABC worked best when taken out of the professional mental health services, as it made mental wellbeing easier to promote, thereby making mental health a more integrated part of public health in general. Bringing messages about the improvement of mental health through meaningful and enjoyable activities (together with others) under the ABC brand made these messages more easily recognisable within and beyond the municipal mental health services. Using ABC as a recognisable framework within diverse settings (voluntary organisations, municipal units, healthcare services, the cultural sector, etc.) and among diverse populations (young families, immigrants, the elderly, etc.) within one municipality potentially supported the building of a health-promoting setting. However, as the previous section showed, this does not happen automatically, and implementing even a seemingly simple and easily adaptable campaign and framework depends on the right timing and requires organisational anchoring, management support, resources, and long-term commitment. If these promoting and inhibiting factors are addressed properly, ABC has the potential to lead to healthier settings. ABC does have some shortcomings, however.

Dooris [30] reminds us that healthy settings initiatives should tackle inequalities and require a relationship with macro-policy. Healthy settings initiatives should "work both upwards and outwards, influencing organizational policies and practices […] and at the same time explicitly addressing broader political, economic and societal factors" (p. 32). This is where ABC lacked an answer, as its focus on enhancing the wellbeing of individuals through meaningful activity largely assumes that everybody has equal means and capacities to do so. As cautionary voices from the interviews pointed out, ABC's simple, positive message does not adequately take socio-economic differences or personal histories into account. It is important to remember here that reducing social inequalities in health is of central importance in the Public Health Act [45]. This issue is something the ABC framework may not be able to address sufficiently. The promoters of ABC acknowledged that they had difficulties reaching and including those who would need mental health promotion the most, due to a lack of means to reach and engage them. Enhanced collaboration with the voluntary, business, or education sector might have helped in finding those who need ABC most, but here the municipalities acknowledged that they fell short (see Table 1).

This shortcoming meant that, with the exception of one of the pilot municipalities, none of the municipalities came close to a whole system or supersetting approach when it comes to building healthy settings [30–32]. These approaches advocate for a broad, holistic, and coordinated strategy when implementing public health campaigns and initiatives. Only in one of the pilot municipalities was the ABC message spread continuously through different channels and by multiple actors towards different populations,ABC actions and measures were implemented within multiple settings, and the course to promote and use ABC in the future appeared stable.

Nevertheless, we argue that ABC offers a simple, recognisable, easily understandable, and transferable message and framework that municipalities can use to create healthier settings for their employees and citizens. It cannot, however, replace systematic public health work or professional mental health treatment, but it can make a valuable contribution.

### Limitations

In this study we deliberately chose to focus on implementation processes rather than on the effects of the ABC campaign. Effectiveness should be a prerequisite for the decision to implement but is thus not investigated in this study. Furthermore, the study employed a convenience sampling strategy in which participants who had experience with ABC and were willing to discuss the realities of the implementation process were recruited. This could have led to an overrepresentation of informants who were positively disposed towards ABC. However, we found the participants to be open, with critical and diverse views of the implementation process.

### Recommendations for further research

Future research on the implementation of ABC should explore how ABC can be implemented to better address socio-economic and cultural differences within populations, particularly among groups that are hardest to reach. Studies should also examine the long-term outcomes of ABC and investigate how to strengthen collaboration with voluntary organisations and other actors. Greater focus on local evaluation is needed to assess both process and impact, enabling municipalities to determine how ABC contributes to mental health promotion.

## Conclusion

This study shows that the successful implementation of the ABC framework depends on a combination of structural, organisational, and contextual factors. Our data show that to implement a public mental health campaign such as ABC, several factors need to be in place. The campaign works best when a municipality already works systematically with public health and has a cross-sectoral network and a dedicated public health coordinator. The flexibility of ABC, its ability to be adapted to diverse settings, populations, and activities, further strengthens implementation by enabling municipalities to tailor the approach to their unique needs and capacities.

At the same time, several inhibitors can limit the success and sustainability of implementation. A lack of systematic public health structures, ongoing organisational instability, overreliance on isolated "fun" activities rather than embedding ABC into everyday structures and long-term work processes, and a lack of financial and human resources, all inhibit the implementation of public health campaigns such as ABC. Furthermore, none of the municipalities had conducted systematic evaluations of their ABC implementation, leaving them without mechanisms for iterative learning, adjustment, or evidence-based decision-making.

When examining ABC's potential for creating healthy settings for mental wellbeing, the framework offers both promise and limitations. It’s simple, positive, and transferable message makes mental health "everybody's business," supporting broader organisational and community engagement. ABC's dual aspect of being both a campaign (hearing) and a framework (doing) is something worth considering for future public health work more broadly.

Yet ABC alone cannot create healthy settings. Its message risks oversimplifying the complex determinants of mental health and may unintentionally place responsibility on individuals without addressing structural or socio-economic barriers. Municipalities struggled to reach those most in need, such as socially isolated individuals, people with fewer resources, or those facing economic hardship. ABC can contribute meaningfully to healthier settings, but it cannot replace the broader strategic, structural, and long-term efforts required to promote mental health and reduce inequalities. When used as part of a comprehensive, well-anchored public health strategy, however, ABC can be implemented in municipalities as a coherent and recognisable framework for enhancing mental wellbeing among both employees and citizens.

## Supplementary Information

Supplementary Material 1.

## Data Availability

The study was conducted in accordance with the ethical principles outlined in the Declaration of Helsinki, ensuring respect for participants’ rights and well-being throughout the research process. All participants received written information about the study and provided informed oral consent prior to the interviews, which were recorded for documentation. The reference number from the local ethics committee, theNorwegian Agency for Shared Services in Education and Research (Kunnskapssektorens tjenesteleverandør – Sikt) for the data protocols/ethical approval and informed consent process for these interviews is 508,035. The research generated data material was not used before in any publication. The data material is not publicly available to guarantee informants anonymity as agreed with the informants and in accordance with the approval of the Norwegian Agency for Shared Services in Education and Research (Kunnskapssektorens tjenesteleverandør – Sikt) for the data protocols/ethical approval. Raw data materials (recordings and transcriptions) have been deleted on 01.01.2026 in accordance with the rules of the Norwegian Agency for Shared Services in Education and Research (Kunnskapssektorens tjenesteleverandør – Sikt) that raw data has to be deleted after the project ended (project end 31.12.2025).
