# Supplementary material for: Mental health promotion in municipal settings – promoters and inhibitors in the implementation of ABC for good mental health in Norway
Source: BMC Public Health. 2026 Apr 30;26:1911. doi: 10.1186/s12889-026-27493-z (PMC13281312; doi:10.1186/s12889-026-27493-z)
Supplement: Supplementary file 1 — Supplementary Material 1. [file 12889_2026_27493_MOESM1_ESM.pdf]

Interview Guide for Public Health Campaigns to Improve Mental Health in the Population with a Focus on ABC for Good Mental Health

Focus of the Interview

- Get an overview of how public health measures and campaigns are implemented in municipalities and organizations.
- Explore opportunities and barriers in implementing public health measures and campaigns in practice, using the ABC framework as an example.
- Increase knowledge and competence about implementation processes for public health measures and campaigns.

Introduction

- How long have you worked here?
- What is your role/work related to mental health in the municipality/population?
- How important do you think prevention is in relation to mental health/illness?

Municipality – Mental Health Work

- Is there more focus on prevention or treatment in mental health work in your municipality?
- What knowledge/competence about preventive mental health work is necessary, and do you have this in the municipality?
- Which other actors do you collaborate with to improve mental health in the population?

Public Health Campaigns in General

- Are you familiar with public health campaigns that aim to improve mental health?
- If yes, which ones?
- In what way did you/your unit/department/municipality use such campaigns? Was such a campaign implemented in the service/municipality?

ABC Implementation Process

- You work in an ABC pilot municipality – do you know why you were chosen as a pilot municipality?
- What training did you receive?
- What support did you receive during the implementation process (from the Directorate of Health, county municipality)?
- Did you receive materials you could use?
- How did you adopt ABC in the service?
- How did the process go, and how far have you come?
- Was there resistance to implementing ABC?
- Has implementing ABC led to extra costs/time investment or to a reduction in costs/time investment?
- Do you in mental health services find the ABC framework useful and relevant for your work?

Facilitators and Barriers

- All municipalities are different – based on the characteristics of your own municipality:
    - What are the biggest facilitators for implementing the ABC campaign?
    - What are the biggest barriers to implementing the ABC campaign?

Collaboration (Partners)

- Which collaboration partners do you work with (think voluntary organizations, businesses, schools, etc.)?
- How did the collaboration go, and did you receive feedback from partners?
- Did you have exchanges/collaboration with other pilot municipalities?

Evaluation and Future

- The pilot phase is now over; how will ABC continue in the service?
- How have you evaluated the ABC campaign?

- What do you recommend other municipalities pay attention to when implementing ABC?
- How does work with the ABC campaign relate to other aspects of the service (think prevention, treatment, clinical work)?
- What is actually new with ABC that you haven't done before?

Ask These Questions Based on Literature Review (if not mentioned by the interviewee)

- In the literature on ABC, we found some barriers and challenges related to ABC – what do you think about these?
  - Time and resource use by personnel – yet another extra task
  - Organizational challenges related to staff turnover
  - How to reach/include vulnerable groups/minorities
  - ABC places responsibility on the individual – do you agree with this?
  - Were there clear evaluation processes?
